# Molecular cloning and expression analysis of dmrt1 and sox9 during gonad development and male reproductive cycle in the lambari fish, *Astyanax altiparanae*

**DOI:** 10.1186/1477-7827-13-2

**Published:** 2015-01-11

**Authors:** Mateus C Adolfi, Ana CO Carreira, Lázaro WO Jesus, Jan Bogerd, Rejane M Funes, Manfred Schartl, Mari C Sogayar, Maria I Borella

**Affiliations:** Department of Cell and Developmental Biology, Institute of Biomedical Science, University de São Paulo, São Paulo, SP Brazil; Department of Physiological Chemistry I, University of Würzburg, Am Hubland, Würzburg, Germany; Chemistry Institute, Biochemistry Department, Cell and Molecular Therapy Center (NUCEL-NETCEM), School of Medicine, University of São Paulo, São Paulo, SP Brazil; Department of Biology, Faculty of Science, Utrecht University, Utrecht, The Netherlands

**Keywords:** Teleostei, Sex differentiation, DMRT1, SOX9, Spermatogenesis

## Abstract

**Background:**

The *dmrt1* and *sox9* genes have a well conserved function related to testis formation in vertebrates, and the group of fish presents a great diversity of species and reproductive mechanisms. The lambari fish (*Astyanax altiparanae*) is an important Neotropical species, where studies on molecular level of sex determination and gonad maturation are scarce.

**Methods:**

Here, we employed molecular cloning techniques to analyze the cDNA sequences of the *dmrt1* and *sox9* genes, and describe the expression pattern of those genes during development and the male reproductive cycle by qRT-PCR, and related to histology of the gonad.

**Results:**

Phylogenetic analyses of predicted amino acid sequences of *dmrt1* and *sox9* clustered *A. altiparanae* in the Ostariophysi group, which is consistent with the morphological phylogeny of this species. Studies of the gonad development revealed that ovary formation occurred at 58 days after hatching (dah), 2 weeks earlier than testis formation. Expression studies of *sox9* and *dmrt1* in different tissues of adult males and females and during development revealed specific expression in the testis, indicating that both genes also have a male-specific role in the adult. During the period of gonad sex differentiation, *dmrt1* seems to have a more significant role than *sox9*. During the male reproductive cycle *dmrt1* and *sox9* are down-regulated after spermiation, indicating a role of these genes in spermatogenesis.

**Conclusions:**

For the first time the *dmrt1* and *sox9* were cloned in a Characiformes species. We show that both genes have a conserved structure and expression, evidencing their role in sex determination, sex differentiation and the male reproductive cycle in *A. altiparanae*. These findings contribute to a better understanding of the molecular mechanisms of sex determination and differentiation in fish.

## Background

The genetic machinery controlling gonad development is widely conserved, where downstream components tend to converge upon the regulation of common effectors. However, comparisons of the sex determination cascades in different organisms show an impressive diversity of ‘master sex-determining genes’ at the top of the genetic hierarchies [1]. In most mammals, it is well known that this process involves the action of a Y chromosomal master gene, named *Sry* [2]. In this case, once the sex is determined, it follows a unique path of development, producing testis or ovary [3, 4]. In contrast, there are many exceptions to this rule in other vertebrates. In fish, gonad development can be influenced by fluctuations of intrinsic factors such as growth and behavior, and by extrinsic factors such as temperature, hormones and exposures to pollutants [5–12]. The medaka fish, like in mammals, possesses an XX-XY sex determination system in which the male is heterogametic, although, like others species of fish, the sex chromosome cannot be morphologically recognized [13]. A gene named *dmy* or *dmrt1bY*, that codes for a protein with the DM domain, was found in the sex determination region of the Y chromosome of medaka. It is a duplicated version of the *dmrt1a* gene [14, 15]. A study in medaka analyzed the main downstream sex determination genes, and showed major differences between mammals and medaka, notably amongst spatial and temporal expression patterns of the canonical signaling pathways, calling into question a strict conservation of regulatory and functional interactions of sexual development genes in vertebrates [16].

The *dmrt1* gene (doublesex/mab-3 related transcription factor-1) belongs to the gene family first found in insects (*doublesex*) and nematodes (*mab-3*), both of which code for a DNA-binding protein with a zinc-finger-like motif, named DM domain [17–19]. The *dmrt1* gene seems to be the only gene whose structure and role are conserved during differentiation and gonad development in males, and which has been found throughout vertebrate evolution [19–26]. It is expressed in germ and Sertoli cells of mice testis, downstream from *Sry* [27]. Mutant *Dmrt1*^−/−^ mice present severe testis problems, showing abnormalities and loss of Sertoli cells function, which may possibly explain the loss of germ cell numbers in these animals. Thus, Dmrt1 is necessary for survival and differentiation of germ and somatic cells in mammals [27].

Some species of turtles and all crocodiles do not have a sex chromosome, with sex being determined by the temperature at which the eggs are incubated [28]. Kettlewell *et al*. [29] showed that expression of *dmrt1* in the genital ridge of turtle embryos was higher in those incubated at lower temperatures, which promotes the formation of male sex. Birds display the ZZ-ZW sex determination system, in which the female is heterogametic (ZW), and the best candidate for male sex determination is Dmrt1, located on the Z chromosome. This gene is expressed in bird embryos of both sexes, but at higher levels in (ZZ) males. Increased expression of this gene at the critical period of sex differentiation leads to testis development, showing a dose-dependent expression for male formation [30, 31].

In fish, all modalities of sex determination have been found, with or without specific sex chromosomes [32]. The *dmrt1* gene was found to be expressed exclusively during the early stages of testis differentiation, but not in the ovary [21, 22, 33]. Induction of sex reversal with androgen in XX Nile tilapia increases the expression of *dmrt1* in the germ-cell-surrounding cells [6]. In *Silurus meridionalis*, two isoforms of *dmrt1* were isolated (*dmrt1a* and *dmrt1b*), with *dmrt1a* being expressed exclusively in gonads, but at higher levels in testis, when compared with ovary. The same pattern was observed for *dmrt1b*, but, besides the gonads, this isoform was also expressed in other tissues, such as kidney and intestine [24].

It is well known that members of the SRY-box (Sox)-family show a role in the formation of gonads. This family encodes a transcription factor which displays a DNA-bind-motif, named SRY-like HMG (high mobility group). *Sox9* is a member of Sox-family that plays an essential role in testis determination besides other functions, e.g. in cartilage formation [34, 35]. This gene seems to be the main effector gene of *Sry* [36]. *Sox9* mutations in an XY organism may lead to bone formation problems, gonad digenesis and sex reversal [35, 37, 38]. The *sox9* gene is conserved in mammals and birds, as well as preserved its structure and function in teleost fish [39–41]. In zebrafish, there are two copies of this gene -*sox9a* and *sox9b*- with *sox9a* expressed in testis and *sox9b* in adult ovary [42]. On the other hand, in medaka, *sox9a* is preferentially expressed in the brain and ovary, and *sox9b* is more expressed in testis than in ovary [43].

*Astyanax altiparanae*, popularly known as lambari, is a neotropical species with ecological and economical importance [44, 45]. *A. altiparanae* was described as a new species in 2000 [46], and little information is available about its reproductive biology and sex differentiation. Recently, we described the morphological alterations of the testis of this species based on the alterations of the germinal epithelium (GE) throughout the annual reproductive cycle [47], but the alterations on the molecular level are not reported. Here, we report the isolation of *dmrt1* and *sox9* sequences and their gene expression patterns during gonad development and in different testes maturation phases in *Astyanax altiparanae*.

## Methods

### Animals

*Astyanax altiparanae* belongs to the Class Actinopterygii, order Characiformes and the family Characidae, which is comprised exclusively of fresh water species, being widely distributed throughout the Paraná River [48].

Larvae and adults of *A. altiparanae* were collected in the Aquaculture and Hydrobiology Station from CESP, located in Paraibuna city, São Paulo State, Brazil. Larvae were raised in our laboratory and collected at 5, 12, 19, 26 and 33 days after hatching (dah). From the 40 dah stage onwards, 3 pools of male and female juvenile gonads (n = 10) were collected each week. Males (n = 3), between 6 and 18 months old, were also collected during the different periods of the reproductive cycle. Adults and juveniles were anesthetized with 0,1% benzocaine and then sacrificed by decapitation. All animal experiments were approved by the Committee of Laboratory Animal Experimentation at University of São Paulo (57/2009).

### Light microscopy

Larvae and juvenile gonads from fish at different phases of development were dissected and fixed in Bouin’s solution for 24 h at room temperature, subsequently dehydrated, embedded in paraffin or historesin (Leica), and then serially sectioned at 3 to 5 μm thickness. The sections were counterstained with hematoxylin & eosin.

### Cloning of *A. altiparanae dmrt1*and *sox9*cDNAs

Tissues of *A. altiparanae* were stored in RNA holder (BioAgency) until RNA extraction. Total RNA was extracted using RNAeasy Mini Kit (Qiagen). First strand cDNA was synthesized with 2 μg total RNA from gonadal tissues in combination with oligo-dT_18_ primer (Fermentas), random primers (Invitrogen) and SuperScript III reverse transcriptase (Invitrogen). Primer sets added to the PCR mixture for the amplification of *A. altiparanae dmrt1* and *sox9* cDNA fragments were designed based on the conserved nucleotide regions of *dmrt1* and *sox9* sequences of several organisms. PCR was performed in a PTC-225 Peltier Thermal Cycler (MJ Research) using the following parameters: 94°C for 30 sec, followed by 35 cycles of 94°C for 10 sec, 50–58°C for 30 sec, and 72°C for 1 min. The PCR was finished by a further incubation at 72°C for 10 min. PCR products were gel-extracted from 1 to 2% agarose gels using QIAquick Gel Extraction Kit (Qiagen) according to the manufacturer’s instructions, and cloned into pGEM-T Easy (Promega), by incubating the reaction with T4 DNA Ligase (pGEM T-Easy Vector System, Promega). Inserts were sequenced on an ABI3700 (Perkin Elmer) sequencer.

To amplify the 5’-and 3’-UTR regions of *dmrt1* and *sox9* genes, we performed the RNA ligase-mediated rapid amplification of 5’ and 3’ cDNA ends using the GeneRacer Kit (Invitrogen) according to the manufacturer’s instructions. All primers used are listed in Table 1.

**Table 1:** **Sequence of primers used in the present study**

Primer	Sequence	Purpose
Dmrt1 I-F	5’-TGCAGAAACCACGGCTTC-3’	cDNA fragment PCR
Dmrt1 I-R	5’-GATGCCCATCTCCTCCTC-3’	cDNA fragment PCR
Dmrt1 II-F	5’-GGCAGTCCCTCCAGTTACAG-3’	cDNA fragment PCR
Dmrt1 II-R	5’-GGGAGGGCTGGTAAAAGTTG-3’	cDNA fragment PCR
Dmrt1 III –R	5’-GRGACAYGTTRTGGCTGGAC-3’	cDNA fragment PCR
Sox9 I-F	5’-GAAGGACCATCCCGACTACA-3’	cDNA fragment PCR
Sox9 I-R	5’-GKGTRTACATKGGCCTCTGG-3’	cDNA fragment PCR
Sox9 II-F	5’-CGAACGTGTTCGGGAACTTA-3’	cDNA fragment PCR
Sox9 II-R	5’-GGCGTGGCTGTAGTAGGAGT-3’	cDNA fragment PCR
Dmrt1 5UTR	5’-AGAGCCACCTGAGCGGCCATGACCC-3’	RACE
Dmrt1 3UTR	5’-GCCTACTACAGCAACCTCTACAATTATCAGCAATACCA-3’	RACE
Sox9 5UTR I	5’-GCACGAGGATCTCTTCCCCCTTAGTTTCTACGCATTTT-3’	RACE
Sox9 5UTR II	5’-GCTCCGCGTTGTGCAGATGCGGGTACTGGT-3’	RACE
Sox9 3UTR	5’-CGCATCTGCACAACGCGGAGCTCAGCAA-3’	RACE
Dmrt1-RT-F	5’-CAGCCTACTACAGCAACCTCTACAAT-3’	qRT-PCR
Dmrt1-RT-R	5’-TGGCTGGACAGACGGCTATC-3’	qRT-PCR
Sox9-RT-F	5’-CCAGCATGGGCGAAGTG-3’	qRT-PCR
Sox9-RT-R	5’-CGTCGGTGGCGTTGGA-3’	qRT-PCR
Ef1a-RT-F	5’-CTTCTCAGGCTGACTGTGC-3’	qRT-PCR
Ef1a-RT-R	5’-CCGCTAGCATTACCCTCC-3’	qRT-PCR
β-Actin-F-Control	5’-CCATCTCCTGCTCGAAGTC-3’	Internal control
β-Actin-R-Control	5’-CACTGCCCATCTACGAG-3’	Internal control

### Sequence analysis

The multiple alignment software Clustal X was employed for alignment of nucleotide sequences and their deduced amino acid sequences, and also to calculate and display the phylogenetic trees using the N-J method. The values represent bootstrap scores of 1,000 trials, indicating the credibility of each branch. The computer programs Clustal X and Boxshade [49] were used to construct the figures.

Accession numbers of the *dmrt1* and *sox9* sequences are as follows: 1) *dmrt1*: *Silurus meridionalis* [EF015487], *Clarias gariepinus* (AF439561), *Danio rerio* [NM_205628.1], *Acipenser transmontanus* [AY057061], *Tetraodon nigroviridis* [AY152820], *Takifugu rubripes* [NM_001037949], *Odontesthes bonariensis* [AY319416], *Xiphophorus maculatus* [AF529187], *Oryzias latipis* [AF319994], *Oncorhynchus mykiss* [AF209095], *Oreochromis niloticus* [AF203489], *Acanthopagrus schlegelii* [AY323953], *Homo sapiens* [AF130728], *Mus musculus* [NM_015826], *Canis familiaris* [XM_846402], *Gallus gallus* [AF123456], *Pelodiscus sinensis* [AB179697]; 2) sox9: *Danio rerio* a [NM_131643.1], *Danio rerio* b [NM_131644.1], *Takifugu rubripes* a [AY277964.1], *Takifugu rubripes* b [AY277965.1], *Monopterus albus* a1 [AF378150.1], *Monopterus albus* a2 [AF378151.1], *Oryzias latipis* a [AY870394.1], *Oryzias latipis* b [AY870393.1], *Oncorhynchus mykiss* [AB006448.1], *Oncorhynchus mykiss* alpha2 [AF209872.1], *Oreochromis niloticus* a [DQ632574.1], *Oreochromis niloticus* b [DQ632575.1], *Gasterosteus aculeatus* [AY351914.1], *Mus musculus* [AF421878.1], *Gallus gallus* [U12533.1]. The nucleotide and amino acid sequences used in the phylogenetic analysis were obtained from GenBank [50].

### Real-time, quantitative PCR (qRT-PCR)

Relative *A. altiparanae dmrt1* and *sox9* mRNA expression levels were assessed by qRT-PCR in the Applied Biosystems 7300 Real-Time PCR System (Applied Biosystems). The primers for qRT-PCR amplification (Table 1) were designed with Primer Express version 3.0 software (Applied Biosystems)*.* The final reaction mixture contained of 3 μL of each primer (0.4 mM), 6 μL of SYBR® Green PCR Master Mix (Applied Biosystems), and 3 μL cDNA reverse transcribed from a standardized amount of total RNA (2 μg). All quantitative reactions were subjected to: 95°C for 10 min followed by 40 cycles at 95°C for 15 sec and 60°C for 1 min, and, at the end, 95°C for 15 sec. Melting curve analysis was applied to all reactions to ensure homogeneity of the reaction product. Potential contamination was assessed by including no-template controls, with no products being observed in these reactions. Dilution curves generated by serial dilutions (1:10) of cDNA were used to calculate amplification efficiencies. Transcript levels of the target genes were normalized against the lambari *ef1a* gene using primers derived from the *D. rerio ef1a* sequence [51]. All the primers used for qRT-PCR were validated and the amplified fragments were sequenced. The ΔCt values presented as means ± standard error of the mean (SEM), were analyzed by one way ANOVA, Tukey’s and Student’s t test. A significance level of P < 0.05 was used for all tests.

## Results

### Sequence analysis of *A. altiparanae dmrt1*and *sox9*cDNAs

**Figure 1:**
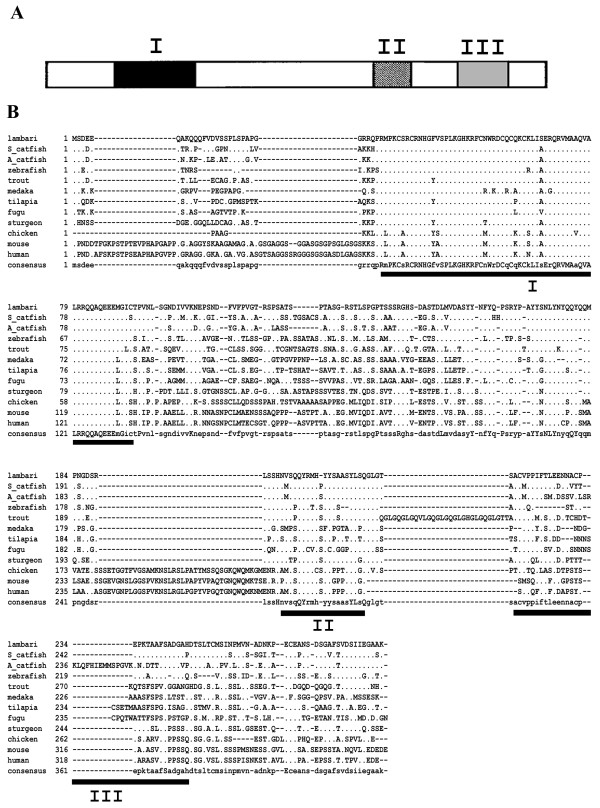
**Deduced aa sequences of** ***A. altiparanae*** **Dmrt1 aligned with those from other vertebrates. (A)** Schematic representation of the Dmrt1 protein structure. **(B)** Regions of high homology are underlined and indicated by Roman numerals: I, the DM-domain; II, the male-specific motif; III, the P/S rich region.

**Figure 2:**
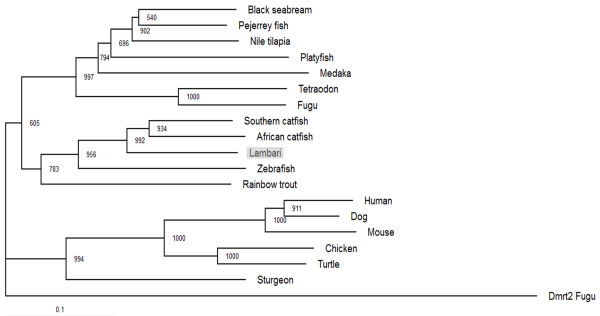
**Phylogenetic tree of Dmrt1 in vertebrates.** The tree was rooted using the fugu Dmrt2 (CAC42780) as outgroup. Numbers on each node are the bootstrap values in thousand runs. The marker length corresponds to a 10% sequence difference.

The isolated *dmrt1* cDNA is 1855 bp long, with an open-reading frame (ORF) of 864 bp, encoding a 287 aa protein (GenBank accession no. KM502983). Lambari Dmrt1 displays all three characteristic domains that are conserved in other vertebrate Dmrt1 proteins (Figure 1). The DNA-binding domain is 62 aa long and shows less divergence than the male-specific domain (20 aa) and the P/S rich region (29 aa) compared with other species. The complete protein sequence of lambari’s Dmrt1 exhibits high similarity with the African catfish (74%) and the zebrafish (74%). A phylogenetic tree was designed by comparing the amino acid sequence of the Dmrt1 of *A. altiparanae* with 17 Dmrt1 sequences from different vertebrate groups using fugu Dmrt2 sequence as the outgroup (Figure 2). The tree shows a high homology of *A. altiparanae* Dmrt1 with the Southern and African catfish, all belonging to the superorder Ostariophysi.

**Figure 3:**
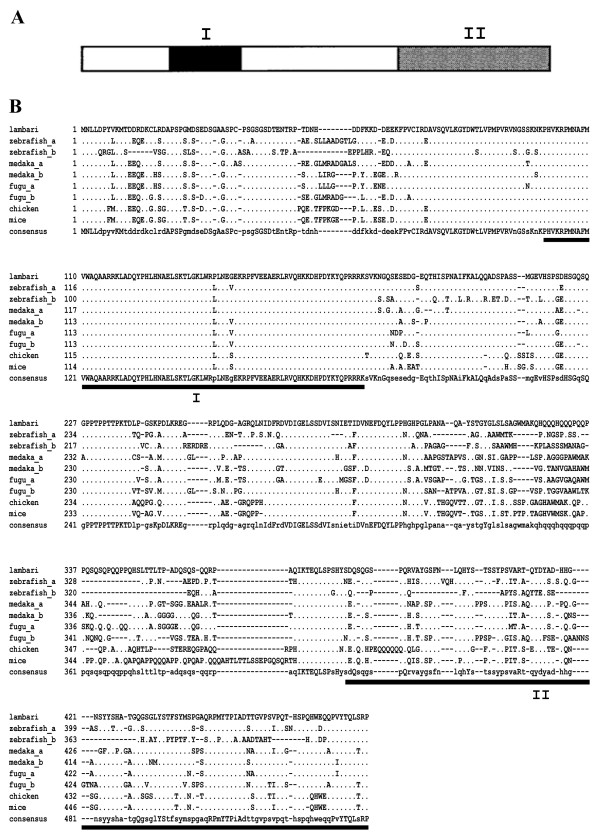
**Deduced aa alignment of** ***A. altiparanae*** **Sox9 with those from other vertebrates. (A)** Schematic representation of the Sox9 protein structure. **(B)** Regions of high homology are underlined and indicated by Roman numerals: I, Sox9-HMG; II, transactivation domain.

**Figure 4:**
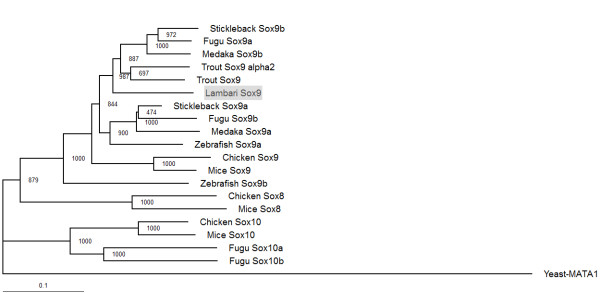
**Phylogenetic tree of Sox E group proteins in vertebrates.** The tree was rooted using yeast-MATA1 (CAA24622.1) as outgroup. Numbers on each node are the bootstrap values in thousand runs. The marker length corresponds to a 10% sequence difference.

The isolated *A. altiparanae sox9* cDNA is 1,533 bp long, and encodes a 483 aa polypeptide, containing the characteristic 78 aa Sox9-HMG domain and the 104 aa transactivation domain (GenBank accession no. KM502984). It shares high similarity with African catfish (78%), sturgeon (74%) and rainbow trout (76%). As shown in Figure. 3, the fragment displays high similarity in the HMG-domain when comparing the Sox9 protein to other clades. The tree constructed in Figure 4 demonstrates that the gene belongs to the Sox9 subfamily, closely related to teleost fish.

**Figure 5:**
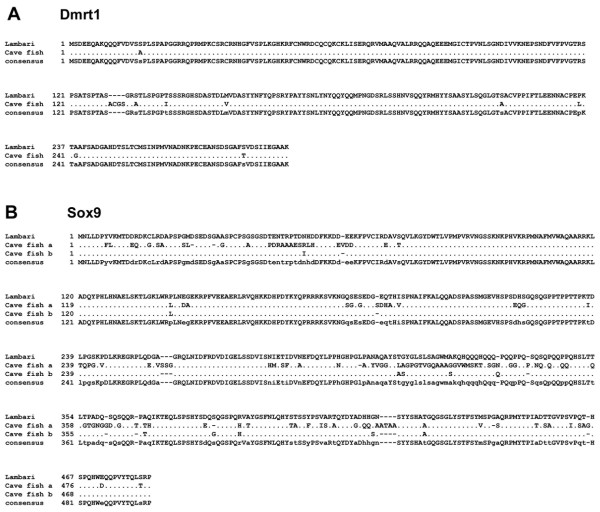
**Comparison of the deduced aa alignments of Dmrt1 (A) and Sox9 (B) proteins of** ***Astyanax altiparanae*** **and** ***Astyanax mexicanus*** **.**

Since the genome of the cave fish, *Astyanax mexicanus*, was released, the sequence of both *dmrt1* and *sox9* of lambari can be compared with another Characidae species. The Dmrt1 protein sequence has 98% similarity between both *Astyanax* species, showing a high conservation of it sequence (Figure 5A). The Sox9 protein of lambari presents 98% identity with Sox9B and 72% with Sox9A of cave fish (Figure 5B).

### Gonad development

**Figure 6:**
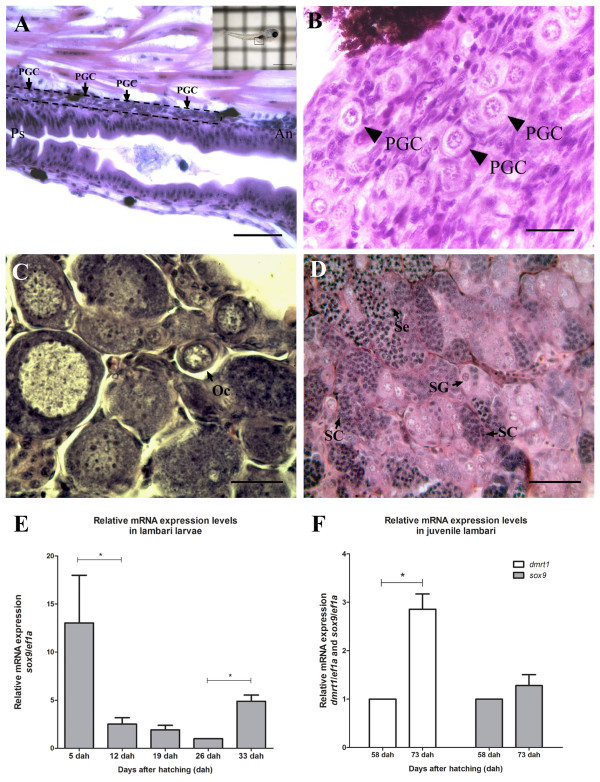
**Gonad development of** ***A. altiparanae*** **(A).** Longitudinal view of a 5 dah larva; scale bar = 50 μm; region indicated by a square in the photography **(B)** Undifferentiated gonad of a 40 dah juvenile; scale bar = 20 μm. **(C)** Juvenile ovary at 58 dah; scale bar = 50 μm. **(D)** Male testis of 73 dah; scale bar = 50 μm. **(E)** *sox9* mRNA expression in larvae of lambari in different stages of development. Values are expressed as arbitrary units of *sox9* mRNA normalized against the expression levels of *ef1a* amplified from the same template, relative to the expression observed in 26 dah. **(F)** *dmrt1* and *sox9* mRNA expression of juvenile lambari. Values are expressed as arbitrary units of both mRNA levels normalized against the expression levels of *ef1a* amplified from the same template, relative to the expression observed in 58 dah. The asterisk indicates significant difference (p < 0.05) after a Student’s t test comparing the developmental stages indicated. An, anterior region of the larva; Oc, oocyte; Ps, posterior region of the larva; PGC, primordial germ cell; SC, spermatocyte; Se, Sertoli cell; SG, spermatogonia.

Following the gonad development of *Astyanax altiparanae* starting at 5 dah, it was possible to find primordial germ cells along the anterior-posterior axis of the genital ridge (Figure 6A). In animals collected before 58 dah only undifferentiated gonads were observed (Figure 6B). At 58 dah, the first sign of gonad formation was found, with the formation of ovaries, where primary oocytes are cleared recognized (Figure 6C). At this time on, undifferentiated gonad was also observed, which later will give rise to the male. The first sign of testis formation was found at 73 dah, presenting the germ cells in cysts, and some in already advanced spermatogenesis stages. However, no sperm was observed in the lumen of the tubules, indicating that these animals were not sexually mature (Figure 6D).

### Gene expression analysis of dmrt1 and sox9

In all larval stages of lambari, no expression of *dmrt*1 was observed. The expression of *sox*9 is relatively high at 5 dah, and is then down-regulated between 5 dah and 12 dah. Expression stays low up to the 26 dah stage, until at 33 dah a significant increase in expression occurs (*p* < 0.05) (Figure 6E). In the juvenile gonad, the mRNAs of *dmrt1* and *sox9* are both detected at 58 dah and increase at 73 dah, with only *dmrt*1 presenting statistical significance (*p* < 0.05) (Figure 6F). These data correlate with the morphological analysis, in which the presence of testicular tissue was found at 73 dah.

**Figure 7:**
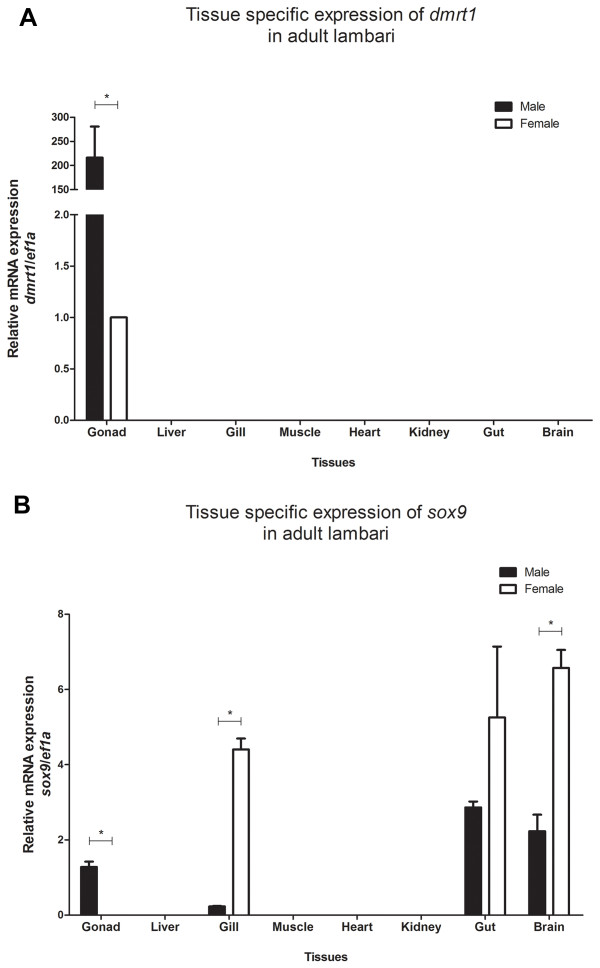
**Tissue specific** ***dmrt1*** **(A) and** ***sox9*** **(B) expression in adult lambari.** Values are expressed as arbitrary units of both mRNA levels normalized against the expression levels of *ef1a* amplified from the same template and relative to the expression observed in female gonads **(A)** and to the average expression observed in gills of male and female **(B)**. The asterisk indicates a significant difference (p < 0.05) after a Student’s t test comparing the expression between male and female gonads.

In adult tissues of *A. altiparanae* we found gonad specific expression of *dmrt1*, with higher expression in testis, displaying more than 200-fold higher expression than the ovary (Figure 7A). *sox9* relative mRNA expression was detected in gills, gut and brain in both males and females sexes. At the level of the gonads, *sox9* expression is male specific. Significant differences in expression levels were observed with higher expression in female than in male tissues, eg brain and gills (*p* < 0.05) (Figure 7B).

**Figure 8:**
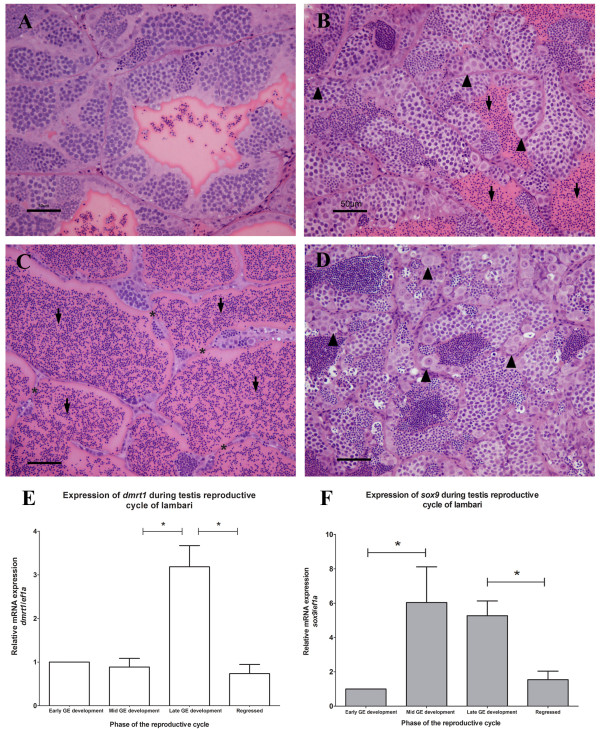
**Testes of** ***A. altiparanae*** **in different phases of annual reproductive cycle. (A)** Early GE development: continuous GE with cysts in different phases of spermatogenesis and low amount of free germ cell in the lumen (arrow); **(B)** Mid GE development: discontinuous GE, with some spermatogonia (arrowhead) and several free germ cells in the tubule lumen (arrow). **(C)** Late GE development: where discontinuous GE (*) could be found with a great amount of free cells immersed in the secretion found in the tubule lumen (arrow). **(D)** Regressed phase: high amount of spermatogonia (arrowhead) and Sertoli cells in the GE. Relative mRNA expression levels of *dmrt1* **(E)** and *sox9* **(F)** during different testes maturation phases. Values are expressed as arbitrary units of both mRNA levels normalized against the expression levels of *ef1a* amplified from the same template and relative to the expression observed during early GE development. Scale bar = 50 μm.

Following the annual reproductive cycle of lambari male as described by Costa *et al.* [47], we collected four phases of testicular maturation: early GE development (Figure 8A), mid GE development (Figure 8B), late GE development (Figure 8C) and regressed (Figure 8D). Analyzing the expression of *dmrt1* we observed a peak of expression in the late GE development phase, but at the other phases the expression is maintained at the same low level (Figure 8E). For *sox9* expression, there is a significant upregulation in the mid and late GE development phases (Figure 8F).

## Discussion

Fish display high diversity of sexual differentiation strategies [3, 52]. Therefore, it is important to study the molecular mechanism of sex determination. Brazil is known for its high abundance of fish species, and the *Astyanax* genus is widely distributed throughout the neotropical territories. However, information about sex determination of this group is still scarce in the literature. In the present study, the complete mRNA sequence of *dmrt1* and *sox9* of *Astyanax altiparanae* were cloned, and their expressions were analyzed during stages of gonad development and throughout the male reproductive cycle.

This group of fish displays high diversity of chromosomal systems, but no sex chromosome was described for *A. altiparanae*, indicating absence of genetic sex determination or homomorphic sex chromosomes [53]. The *doublesex and mab-3 transcription factor 1* gene was shown to be conserved during evolution, as described in mammals, reptiles, amphibians, fish and invertebrate [19–24, 54]. Analyzing the DM domain structure and the phylogenetic tree of the deduced Dmrt1 amino acid sequence of lambari, we found that its sequence is similar to species that belong to the Superorder Ostariophysi. The Ostariophysi is composed by four orders: Characiformes (tetras, piranhas), Siluriformes (catfishes), Cypriniformes (carps, zebrafish) and Gymnotiformes (electric fish). The DM domain of lambari varies in only one amino acid, when compared with other vertebrate species (A-S). Interestingly this same amino acid is changed in fugu (A-V). However, this amino acid change is conservative in fugu, but this is not the case for *A. altiparanae*. Biochemical experiments have to be done to see if this single change has an influence on the DNA-binding properties of the protein.

The *dmrt1* gene is not only characterized by its DM-domain, but also by its male-specific region. Two types of DSX genes are present in *Drosophila*, namely the male type (DSX_m_) and the female type (DSX_f_). The male-specific motif is present only in the DSX_m_. The male specific motif has also been characterized by molecular cloning in vertebrates [21, 24, 33]. By molecular cloning of lambari *dmrt1*, we collected additional evidence of the evolutionary conservation of this domain, since only two amino acid changes were observed when compared with the African catfish sequence.

More than one isoform of *dmrt1* in fish species have been described [24, 25]. Liu *et al*. [24] described an alternative splicing isoform of *dmrt1,* with the DM domain being similar for both isoforms. Although only one isoform was isolated in the present work, it is possible that more isoforms may be found in *Astyanax altiparanae*.

The *sox9* gene is another sex determination related gene, which has been well described in mammals. However, this gene is also important for cartilage formation [34, 35, 55]. This gene also contains a DNA binding domain, known as HMG domain, which is characteristic of the Sox family [56, 57]. By sequence comparison, we found that the *sox9* gene from *Astyanax altiparanae* belongs to the SoxE subfamily. Analysis of gene sequences and of the Sox9 phylogenetic tree shows that *Astyanax altiparanae* clusters in the basal teleost fish, confirming the previously described morphological data. Another characteristic of the Sox9 protein is the presence of the transactivation domain in the C-terminal region. This domain is also conserved being rich in proline, glutamine and serine. Mutations in this transactivation domain lead to sex reversal in humans, suggesting that mutations probably inhibit transactivation of *sox9* downstream genes in mammals [58]. Analyzing the transactivation domain of lambari, we show a high degree of similarity with other teleost fish, particularly, with the *sox9b* gene copy.

It has been proposed that gene duplication facilitates the evolution of gene functions, via mechanisms of neofunctionalization and subfunctionalization [59]. Some human gene families demonstrate the history of two rounds of gene duplication during early vertebrate evolution, and fish genomes have often two co-orthologs for many human genes, as a result of a third round of genome duplication that occurred at the base of the teleost radiation [60]. Cresko *et al*. [61] described in zebrafish that the combined expression pattern of the two *sox9* genes approximately corresponds to that of the single *Sox9* in mouse, which is indicative of a partitioning of an ancestral function. In this work we isolated only one copy of *sox9* in lambari, derived from testis samples, which also showed male specific expression in gonads. The genome of *A. mexicanus* provides us a predicted sequence of a second copy of the *sox9* gene, and together with our phylogenetic analyses, the *sox9* gene of lambari shows higher identity with other teleost *sox9b* genes. However, the presence of another *sox9* gene copy in lambari can only be confirmed by isolation and characterization of this gene in this species. The *sox9a* and *sox9b* of zebrafish are more divergent in comparison to other teleost sequences, and even lambari being closer phylogenetically to zebrafish, the Sox9 sequence does not cluster together with the zebrafish sequence. But the sequence of Sox9 from lambari still clusters in the group of basal teleost, going along with morphological analyses [62].

The pattern and timing of gonad differentiation and sex determination have been studied in fish [63–68]. In most gonochoric teleost species, the ovary develops first while in male the gonad remains undifferentiated and a few days, weeks or even months later the formation of testis occurs [67, 68]. Apparently, this pattern is observed during the development of lambari gonads, with the first sign of ovary (at 58 dah) being observed in female. In males the gonad remains undifferentiated. Only two weeks later the testis formation was observed in males (at 73 dah). However, the testes showed some cysts in advanced stages, indicating that the formation of the organ occurred sometime before that observed. The origin of germ cells occurs independently from the gonadal tissue, and, during development, these cells migrate to the genital ridge [69–71]. The histological analysis of gonads during lambari development showed that at 5 dah the germ cells are already located in the genital ridge, where the future gonad will be located.

During gonad development, the exact timing of *dmrt1* and *sox9* expression and sex determination varies between species, but, in general, their expression is correlated to the formation of testis [72]. In zebrafish and medaka, the onset of *dmrt1* expression occurs in the first days after hatching (10 dah), and in medaka this gene is the first that is differentially expressed, when male and female are compared [73, 74]. The *dmrt1* gene has been shown to be the main sex determination gene, being critical for male determination [75]. In lambari, the expression of *dmrt1* was just observed in the juvenile gonads where no testis structure was observed (at 58 dah), indicating that the role of *dmrt1* in the testis is most likely prior to visible sex differentiation.

In amniotes, the expression of *dmrt1* is restricted to gonads, being more highly expressed in testis than in ovary. Our data go together with other fish species, where the expression of *dmrt1* is apparently restricted to gonads, similarly to amniotes. The testicular form of *sox9* is an upstream gene in the sex differentiation cascade being conserved in vertebrates [57]. In the gonad of lambari, the *sox9* expression is male-specific. However, our data show expression of *sox9* in other adult tissues of lambari not only in gonad, a result that has also been observed in zebrafish [42, 74]. However, the higher expression of *sox9* observed in gut of both males and females and the higher expression in female gills when compared to male gills, are totally uncommon in fish (Table 2). In chicken and *Lepidochelys olivacea* turtle, *Sox9* expression was identified in undifferentiated gonads, being upregulated during testis formation, and downregulated when it differentiates into ovary [76, 77]. The function of *sox9* in fish gonad differentiation remains unknown. In zebrafish, a high expression of the *sox9a* variant has been shown during gonadal differentiation to male [74]. In medaka no *sox9* expression was shown in the early developing gonad, when comparing male and female embryos. This shows that the role of this gene is not related to the early sex determination in medaka [78]. In lambari, there is also no difference of *sox9* expression before and after the differentiation of testis, indicating that *dmrt1* is apparently more crucial for this role than *sox9*. However, the expression level of *sox9* is already observed in the larvae of lambari, showing higher levels at 5 dah, being down-regulated at 12, 19 and 26 dah. Since the expression levels of *sox9* was determined from the whole larvae body, those early expression levels are not necessarily related to gonad formation or sex determination. To confirm if the expression pattern of *dmrt1* and sox9 in adults and during the development of the gonad is directly related to sex determination and differentiation, future functional experiments have to be performed.

**Table 2:** **Gene expression of** ***sox9a*** **and** ***sox9b*** **in adult tissues of different teleost specie**

	Liver		Gills		Muscle		Heart		Kidney		Gut		Brain		Testis		Ovary	
	sox9a	sox9b	sox9a	sox9b	sox9a	sox9b	sox9a	sox9b	sox9a	sox9b	sox9a	sox9b	sox9a	sox9b	sox9a	sox9b	sox9a	sox9b
*Astyanax altiparanae*	-		++		-		-		-		++		++		+		-	
*Oncorhynchus mykiss* ^*^	+		N.R.		N.R.		+		N.R.		N.R.		+		+		+	
*Oryzias latipes*	-	-	+	+	-	-	-	+	-	-	N.R.		++	++	+	+++	+++	-
*Danio rerio*	-	-	+	+	+	-	+	-	+	+	N.R.		++	+	++	-	-	+++

N.R., data not reported.

^*^Data of rainbow trout *sox9.*

The morphology of *A. altiparanae* testis shows that during the annual reproductive cycle the male gonad passes through complex changes in the structure of the germinal epithelium [47]. Our data show that the expression pattern of *dmrt1* and *sox9* also change during the process of gonad maturation, especially at the late GE development phase, where the lumen was filled with spermatids and spermatozoa. In tilapia with *dmrt1*-deficient testes, a significant testicular regression was observed including deformed efferent ducts, degenerated spermatogonia or even loss of germ cells, and proliferation of steroidogenic cells [79]. In medaka, a loss-of-function mutation in *dmrt1* leads to a complete male-to-female sex reversal during the phase of maintenance of the gonad [80]. Expression analyses of *dmrt1* in rainbow and pejerrey during the different spermatogenesis stages showed a similar situation in lambari, where *dmrt1* expression is extremely decreased after spermiation. Analysis of medaka *sox9b* reveals that this gene has a important role in the germ cell maintenance in males and females, which could explain the differences of lambari *sox9* gene expression throughout the testis maturation cycle [81].

## Conclusions

The lambari *dmrt1* and *sox9* gene sequences are conserved and are closely related to sequences of other basal teleosts. Expression analysis of both genes revealed testis specificity, indicating that these genes display male-specific functions in the adult lambari, probably in gonad maintenance. The expression patterns of *dmrt1* and *sox9* change throughout the reproductive cycle of male, with a significant down-regulation after spermiation, indicating a possible role in spermatogenesis. During gonad formation, the differentiation of the ovary begins earlier than testis, with an upregulation of *dmrt1* at 78 dah, when the first testis structures can be recognized. This indicates that the male sex determination period occurs before this stage. In larvae, no expression of *dmrt1* is observed, but the *sox9* gene expression is downregulated at 12 dah, and is upregulated at 33 dah. Future functional experiments can help to confirm the role of *dmrt1* and *sox9* genes in the sex determination, differentiation and male reproductive cycle in *Astyanax altiparanae*.

## Abbreviations

DMRT1: Doublesex/mab-3 related transcription factor-1
DSX: Doublesex
EF1a: Elongation factor-1 alpha
GE: Germinal epithelium
HMG: High mobility group
ORF: Open reading frame
SOX9: SRY-box 9
SRY: Sex determining region of Y.
